# Canal Cristae Growth and Fiber Extension to the Outer Hair Cells of the Mouse Ear Require *Prox1* Activity

**DOI:** 10.1371/journal.pone.0009377

**Published:** 2010-02-23

**Authors:** Bernd Fritzsch, Miriam Dillard, Alfonso Lavado, Natasha L. Harvey, Israt Jahan

**Affiliations:** 1 Department of Biology, University of Iowa, Iowa City, Iowa, United States of America; 2 Department of Genetics and Tumor Cell Biology, St. Jude Children's Research Hospital, Memphis, Tennessee, United States of America; University of Oldenburg, Germany

## Abstract

**Background:**

The homeobox gene *Prox1* is required for lens, retina, pancreas, liver, and lymphatic vasculature development and is expressed in inner ear supporting cells and neurons.

**Methodology/Principal Findings:**

We have investigated the role of *Prox1* in the developing mouse ear taking advantage of available standard and conditional *Prox1* mutant mouse strains using *Tg(Pax2-Cre)* and *Tg(Nes-Cre)*. A severe reduction in the size of the canal cristae but not of other vestibular organs or the cochlea was identified in the E18.5 *Prox1^Flox/Flox^; Tg(Pax2-Cre)* mutant ear. In these mutant embryos, hair cell differentiated; however, their distribution pattern was slightly disorganized in the cochlea where the growth of type II nerve fibers to outer hair cells along *Prox1* expressing supporting cells was severely disrupted. In the case of *Nestin-Cre*, we found that newborn *Prox1^Flox/Flox^; Tg(Nestin-Cre)* exhibit only a disorganized innervation of outer hair cells despite apparently normal cellular differentiation of the organ of Corti, suggesting a cell-autonomous function of *Prox1* in neurons.

**Conclusions/Significance:**

These results identify a dual role of *Prox1* during inner ear development; growth of the canal cristae and fiber guidance of Type II fibers along supporting cells in the cochlea.

## Introduction

The mammalian inner ear is composed of the cochlea that mediates the auditory function, and the vestibule that mediates the gravitational and angular acceleration sensing. In mammals, six epithelial sensory patches found in the cochlear and vestibular regions of the inner ear mediate auditory and vestibular functions: the organ of Corti is the sensory patch found in the cochlea and three cristae and two maculae are the sensory patches of the vestibule. Each of these sensory patches includes mechanosensory hair cells and non-sensory supporting cells. Both of these cell types originate from epithelial progenitors that become specified as prosensory precursors. According to their position in the ear, these prosensory patches will give rise to the definite vestibular or cochlear sensory patches. Cells in those sensory patches ultimately assume final fates as either hair cells (e.g., inner and outer hair cells in the cochlea) or a variable number of non-sensory supporting cells (distributed between hair cells). While the molecular machinery governing the development of hair cells has received much attention [1], [2] far less is known about the molecular basis of cell fate decision in supporting cells [3], [4]. In the mammalian cochlea, at least five unique types of supporting cell can be identified: Pillar cells, Deiter's cells, Hensen cells, Claudius cells and inner sulcus cells [3], [5].

We and others have proposed that the development of the vertebrate ear sensory epithelium shares certain similarities with the development of the sensilla in insects [6], [7], [8]. In *Drosphila*, the homeobox gene *prospero* plays important roles in cell fate decision during glia, sensory sensilla, and eye development [9], [10], [11], [12], [13], [14]. *Prox1*, the vertebrate counterpart of *prospero* [15] is expressed in several murine cell types where its function is essential for proper development and differentiation [15], [16], [17], [18], [19], [20], [21], [22], [23], [24].

Interestingly, in addition to the developing retina [15], [20] and spinal cord [25], *Prox1* expression was also identified in another sensory organ; i.e., the developing ear of zebrafish [26], chicken [27], and mice [28], [29]. By taking advantage of available standard and conditional *Prox1* mouse mutant strains [30], [31], we have now determined that *Prox1* is an important new player during the development of the mammalian vestibular and auditory systems. We demonstrate that in the canal cristae, lack of *Prox1* function affects the overall growth of these vestibular sensory epithelia. In contrast, in the cochlea, absence of *Prox1* disrupts stereotyped cellular organization and fiber guidance of Type II neurons apparently in a cell autonomous fashion.

## Methods

### Mice

*Prox1^+/LacZ^*, *Prox1^flox/flox^*, *Atoh1*, and *Tg(Pax2-Cre)* and *Tg(Nes-Cre)* mice have been previously reported [30], [31], [32], [33], [34], [35], [36]. The developmental stage of mouse embryos was determined by considering noon of the day the vaginal plug was detected in the pregnant dam as E0.5. All of the mouse experiments were approved by the Creighton University, University of Iowa, and St. Jude Children's Research Hospital Animal Care and Use Committees.

### Detection of β-Galactosidase Activity

To detect β-gal activity, ears were dissected and X-gal staining was performed as described previously [37]. Whenever required, we enhanced the X-gal reaction using 2-photon photoactivation on whole mounts and sections [38]. In addition, we ran some ears without fixation to avoid any quenching of the β-galactosidase activity. Stained ears were mounted flat or alternatively, they were embedded in epoxy resin, sectioned (20 µm) and imaged using a compound lightmicroscope (Nikon Eclipse 800) and captured using a Coolsnap camera and Metamorph software. Some ears were processed for transmission electron microscopy and viewed in a Hitachi TEM as previously described [39]. Unfortunately, use of either *Tg*(*Pax2-Cre)* or *Tg(Nes-Cre)* leads to early postnatal lethality; therefore, we were not able to analyze the conditional mutant ear beyond P1.

#### *Prox1* in situ hybridization

Whole mount in situ hybridization was performed using a riboprobe as previously described [15].

### Immunohistochemistry

Primary antibodies were rabbit anti–β-gal (ICN), rabbit (Covance Research Products) anti–mouse Prox1 (Promega), rat anti–mouse β-tubulin (Sigma), Hoechst nuclear stain (Sigma), Myo VII (gift of T. Hasson, San Diego), Sox2 and BDNF (Invitrogen). Secondary antibodies were Alexa 488, 543, and 634–conjugated donkey anti-rabbit (Molecular Probes), Cy3-conjugated donkey anti–guinea pig (Jackson ImmunoResearch Laboratories), and Cy3-conjugated donkey anti-rat (Jackson ImmunoResearch Laboratories) were used predominantly on whole mounted microdissected sensory epithelia [40]. Sections and whole mounts were imaged using a confocal system (Zeiss LSM 510 or Leica SP5). Images were assembled into plates using CorelDraw software. Size of sensory epithelia was measured using ImagePro software on fully calibrated confocal images. PTI lipophilic tracers (NV Maroon) were used for afferent and efferent fibers [41]. Briefly, dyes were inserted into central targets or as small local injections and the fibers were filled with the diffusible dye, epithelia were microdissected and viewed with a confocal system (Zeiss LSM 510 or Leica SP5).

### Quantification

In order to evaluate the qualitative effects of lack of *Prox1* function on the growth of the vestibular epithelia we measured the length of the anterior canal crista and the utricle using the calibration setting of the Zeiss LSM 510 system in six flat mounted vestibular organs of *Prox1^flox/flox^*; Tg(*Pax2-Cre)* (E18.5 mutant) and *Pax2-Cre* (E18.5 control). Differences were evaluated for significance suing a *T-test*. We also counted the number of hair cells using Myo VII immunocytochemistry to identify hair cells and Hoechst nuclear staining to label the nuclei in three of these vestibular areas of control and mutant mice. Counting was done on flat mounts of three anterior canal cristae by grabbing a confocal stack at 6 µm interval (slightly wider than the average nuclear diameter to avoid double counting). Shrinking or other counting artifacts should be equal but this procedure will slightly underestimate the total number of hair cells [42], [43]. A non-parametric rank correlation test was used to assess statistical significance of cell counts.

## Results

### Prox1 Expression in the Developing Inner Ear

Previous work using immunohistochemistry reported that Prox1 expression in the inner ear starts around E11.0 in three vestibular sensory patches and around E11.5 is highly expressed in the canal cristae and saccule of the sensory epithelia and weakly in the utricle [28]. Expression in the cochlea starts at around E14.5 in Pillar cells, Deiter's cells and outer hair cells, and also extends weakly to nonsensory parts of the ear [29].

**Figure 1:**
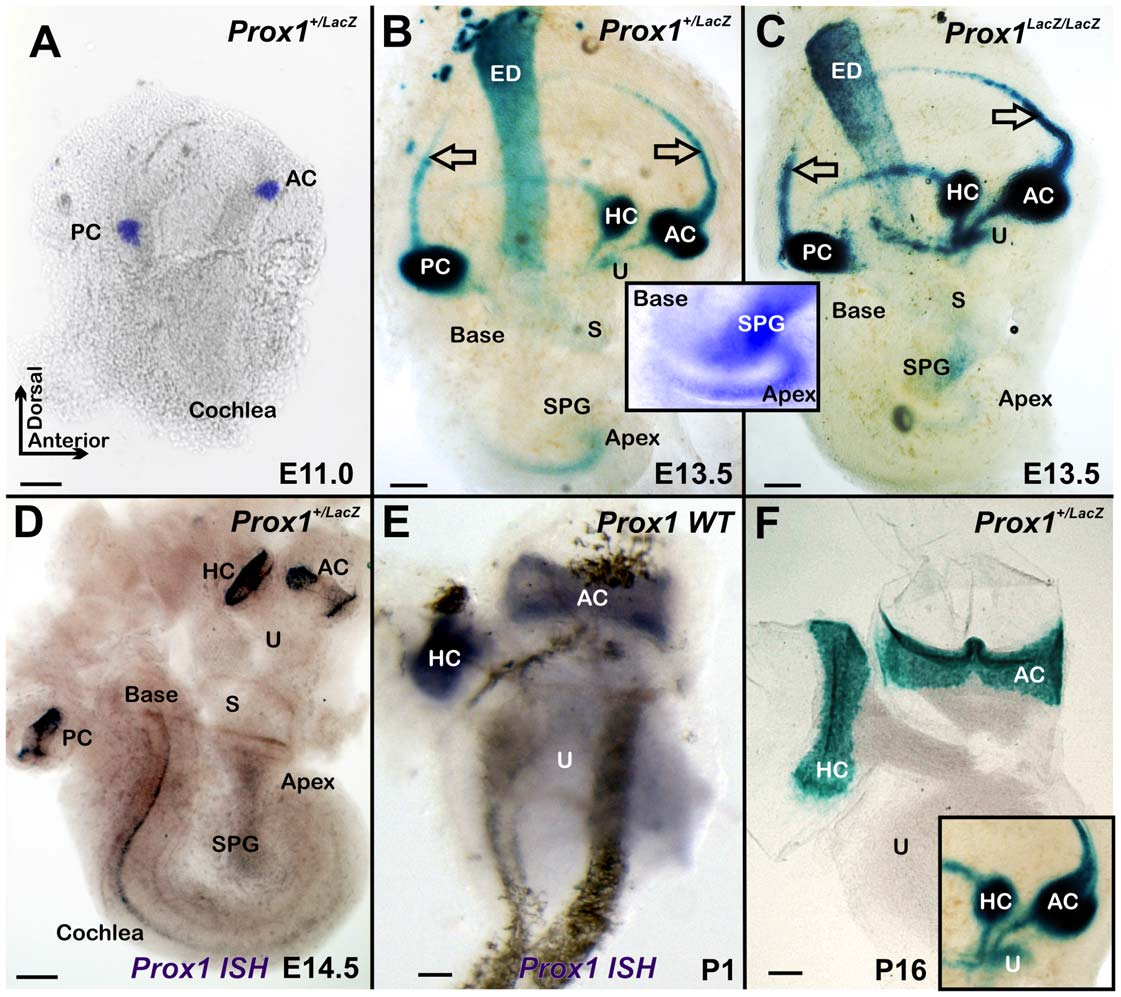
The early onset of Prox1 expression is revealed by β-galactosidase expression and in situ hybridization. Whole mount β-galactosidase histochemcial reaction using X-Gal was performed in *Prox1* heterozygous and nullizygous embryos. A. Starting at E11.0, a progressive upregulation of *Prox1* is seen in the anterior (AC) and posterior (PC) canal cristae. B. By E13.5, expression is also detected in the horizontal canal crista (HC), the striolar region of the utricle (U), the canals and the endolymphatic duct (ED); expression in the saccule is barely detected (S). In the cochlea, upregulation of β-galactosidase expression is detected in the apex and decreases toward the base. Arrows indicate expression in anterior and posterior canal with their expression. C. Expression of β-galactosidase is identical in heterozygous and nullizygous mice with the exception that the signal is stronger in nullizygous mice. Faint β-galactosidase expression is also detected in the delaminating spiral ganglion neurons (SPG; C and insert in B,C). D. In situ hybridization shows at E14.5 expression in the canal cristae and the cochlea, but indicates a more prominent upregulation in the base at this stage. Only spiral ganglion sensory neurons are faintly positive for *Prox1* in situ (SPG in D). E,F At postnatal stages, *Prox1* expression remains in the canal cristae as revealed by in situ hybridization for *Prox1* mRNA or X-Gal reaction, but does not show the extensive expression in the non-sensory parts of the canals as in earlier stages (insert in F). Bar, 100 µm.

In order to precisely compare the profile of *Prox1* expression with the well known onset of hair cell proliferation [40], [44] and differentiation [45], [46] we took advantage of an available *Prox1* heterozygous strain in which the β-galactosidase reporter gene was inserted in frame into the *Prox1* genomic locus [31]. As shown in Fig. 1A, at E11.0 *Prox1* expression was restricted to two X-gal positive patches corresponding to the anterior and posterior canal cristae. Two days later, an additional third patch of expression was seen in the region corresponding to the horizontal crista (Fig. 1B). Around E13.5 *Prox1* expression also starts to be detected in what appears to be the striola region of the utricle and is barely detected in the saccule (Fig. 1B). It is only at around this stage that *Prox1* expression starts to be detected in the cochlea where *Prox1* upregulation begins broadly in the apex and expands toward the base (Fig. 1B). *Prox1* expression is not restricted to sensory epithelia but is also found in the forming canals and the endolymphatic duct (Fig. 1B). In addition, *Prox1* expression starts in the spiral ganglia around that time (Fig. 1C, insert). *Prox1* in situ hybridization detects signal in the canal cristae but in the organ of Corti of the cochlear duct only at E14.5 (Fig. 1D). As indicated by X-gal staining (Fig. 1F) and in situ hybridization (Fig. 1E), *Prox1* expression remains in the newborn canal cristae but is lost in the non-sensory part of the canal (insert in Fig. 1F).

### Canal Cristae Are Smaller in Prox1-Null Embryos

Next, and in order to identify possible functional roles of *Prox1* during the development of the ear, we characterized the inner ear of E14.5 *Prox1*-null embryos [31]. It was previously reported that *Prox1*-null embryos die at around E14.5 [31]. In agreement with the lack of Prox1 expression in developing sensory neurons at early developmental stages, no obvious phenotypic alterations were identified in the *Prox1*-null ears prior to E14.5 (Fig. 1B,C). This data indicated that Prox1 activity is not required for sensory neuron differentiation at these early stages.

**Figure 2:**
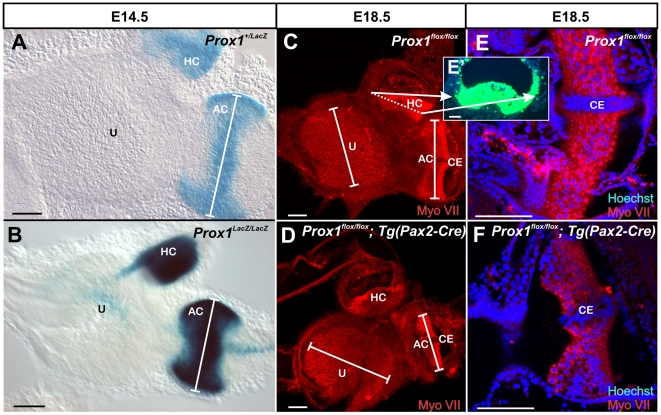
Effects of Prox1 loss-of function in the vestibular epithelia. A. X-gal staining of E14.5 *Prox1* heterozygous embryos reveals β-galactosidase activity in the anterior (AC) and horizontal (HC) parts of the canal cristae. B. Although morphologically normal, a reduction in the size of the crista epithelia is detected of *Prox1*-null littermates (white bar in the AC); gravistatic sensors such as utricle (U) show only transient *Prox1* expression and no apparent reduction in size. C, E. Hair cells are revealed using antibodies against Myo VII in a normal E18.5 *Prox1^flox/flox^* conditional embryo. Note absence of imunoreactivity in the cruciate eminence (CE) of the anterior canal crista. E′. As shown by 2-photon activation, at this later stage, *Prox1* expression is high in supporting cells, but is also found in hair cells of the canal cristae as well as outside the sensory epithelium. Dotted line in B indicate the plane of sections through the horizontal canal crista, white arrows align lateral walls of the whole mount with the section. E,F. Despite the overlap of some Prox1 expression with hair cells in the canal cristae there is no morphologically obvious defects in hair cell differentiation other than reduced intensity of Myo VII staining are observed in *Prox1^ flox/flox^; Tg(Pax2-Cre)* as compared to *Prox1^flox/flox^* littermates. However the reduction in size of the anterior canal crista (AC) is becoming more obvious at this late stage (C–F). CE-Cruciate eminence. Bar, 100 µm.

**Figure 3:**
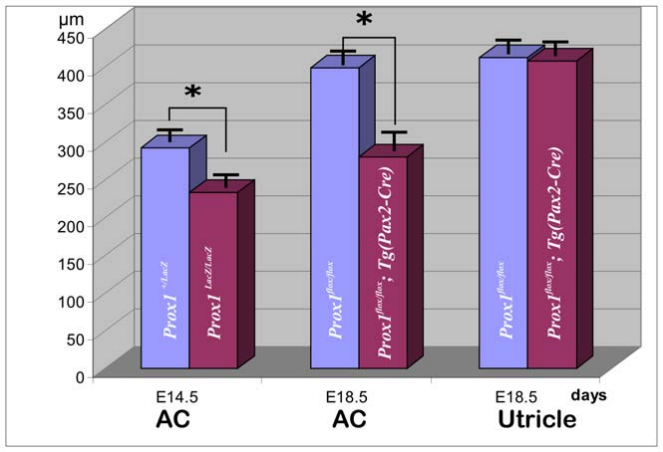
Prox1 inactivation reduces the size of the anterior crista. As measured at E14.5, the length of the anterior cristae (AC) of *Prox1* mutant embryos is 20% reduced when compare vs. that of wild-type littermates. The size reduction is 30% when compared with the size of E18.5 *Prox1^ flox/flox^; Tg(Pax2-Cre)* mutant embryos. No significant changes in the length of the utricle were observed. Asterisks indicate a level of significance (p<0.05; *t-test*).

As indicated above, high levels of *Prox1* expression are detected in the developing canal cristae (Fig. 1). In agreement with this expression and as revealed by X-gal and Myo VII stainings [an early marker of hair cell differentiation; [47]], the size of the anterior canal cristae (AC) was clearly reduced in E14.5 *Prox1*-null embryos (Fig. 2A–D, Fig. 3). The posterior canal cristae (PC) was similarly affected (data not shown) and the horizontal canal cristae (HC) was not as affected (Fig. 2A–D). In addition to the high level of expression in the canal cristae, X-gal staining of E14.5 *Prox1* heterozygous and nullizygous embryos confirmed that *Prox1* expression was only transient and weak in the utricle (Fig. 2A,B) and almost not detectable in the saccule (Fig. 1C). In situ hybridization verified that a weak but detectable signal persisted in the utricle at least until P1 (Fig. 1F) as previously described [29]. We determined that on average (N = 6), the size of the anterior canal cristae in *Prox1*-null embryos was 20% smaller (p<0.05; *t-*test) than in their heterozygous littermates (Fig. 3) (no differences in size were found between wild-type and *Prox1* heterozygous littermates; data not shown). We also counted the number of hair cells and found that the anterior canal cristae of *Prox1*-null embryos had only about 605 (+−65) hair cells compared to the control littermate that had about 913 (+−78) hair cells (p<0.05).

To confirm and expand this observation indicating that removal of *Prox1* activity affects the size of the vestibular sensory epithelia, we took advantage of a previously generated *Prox1* conditional knock-out strain [30] to remove *Prox1* activity from the inner ear in a time and tissue specific manner. To this end, *Tg*(*Pax2-Cre)* transgenic mice [33] were used to delete *Prox1* from E9.0 onward in all cells of the ear, including all hair cells and sensory neurons [48]. Using this approach we also expected to overcome the early embryonic lethality of standard *Prox1*-null embryos [31]. Analysis of *Prox1^flox/flox^;Tg*(*Pax2-Cre)* conditional mutant embryos at E18.5 identified phenotypic alterations similar to those described in the E14.5 *Prox1*-null embryos; e.g., the size of the anterior cristae was significantly reduced (30% N = 6; p<0.05; *T-test*) (Fig. 2D, Fig. 3). Despite this size reduction, the overall shape and morphology of the cristae, and the formation of the non-sensory cruciate eminence (CE) were not affected in these mutant embryos (Fig. 2D, F). As indicated by Myo VII staining, no obvious gross morphological alterations were detected in the development and distribution of the vestibular hair cells of the canal cristae at these later stages (Fig. 2D, F). No obvious alterations in the distribution and morphology of supporting cells (indicated by Hoechst stained nuclei), or in the size of the utricle were identified in these conditional mutant embryos (Figs. 2,3). In E18.5 *Prox1* heterozygous animals, expression as revealed with 2 photon photoactivation of the β-galactosidase reaction product [38], is found throughout all supporting cells of the canal cristae. In agreement with a recent report [29], at this stage Prox1 expression was also detected in some hair cells and non-sensory cells adjacent to the canal cristae (Fig. 2E′).

In summary, these initial results revealed that removal of *Prox1* function from the developing ear resulted in a significant reduction in the size of the canal cristae.

### Lack of Prox1 Function Results in Hair Cell Misalignment and Disrupted Type II Spiral Ganglion Cell Guidance

**Figure 4:**
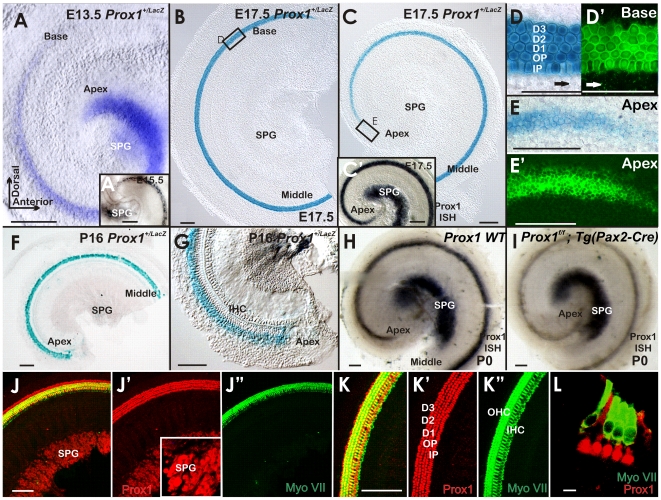
Prox1 expression in the cochlea is biphasic. **A.** As shown by X-gal staining, at E13.5 *Prox1* expression in the cochlea is higher in the apex and gradually faints toward the base; with limited expression in delaminating sensory neurons (SPG in A, A′). B,C. Expression is later on found throughout the organ of Corti. C. This elevated expression has not yet reached the undifferentiated apex (C, E,E′) which is confirmed by *Prox1* in situ hybridization (C′) that also indicates Prox1 expression in the spiral ganglion (SPG; C′). D, D′. Whole mount analysis, including 2 photon activation of the β-galactosidase reaction product (D′) show that near the base the expression of *Prox1* is nearly exclusive found in the five supporting cells of the lesser epithelial ridge (three rows of Deiter's cell, D1–3; two rows of pillar cells (IP, OP) with limited expression in some outer hair cells (arrow C′) and inner phalangeal cells (arrows in D, D′). E,E′. In the apex the expression of *Prox1* is not restricted to just five rows of cells, reflecting the immature state of the apex with incomplete convergent extension and possible expanded expression of Prox1. F,G. Expression in supporting cells stays on in young adults and there is a faint expression in spiral ganglion cells (SPG; F). No labeling is found around inner hair cells (IHC) in postnatal animals (F–I). H, I. *Prox1* expression was verified using in situ hybridization in newborn wildtype and *Prox1^ flox/flox^; Tg(Pax2-Cre)* conditional mutants. Note the prominent presence of the in situ signal in sensory neurons and the slight reduction of the overall signal in the organ of Corti in the conditional null mice (I) that is possibly related to the disorganization of supporting cells (see Fig. 5). The *in situ* hybridization will detect the full length and the conditionally truncated and non functional mRNA of Prox1. Immunocytochemistry on whole mounts (J,K) and sections (L) verifies the data obtained with X-Gal reaction and in situ hybiridization and reveals a prominent expression in supporting cells (J,J′, K,K′ L) and spiral ganglion neurons (SGN, J, J′). Myosin VII (Myo VII) stain hair cells (J″, K″, L) but not supporting cells. Bar, 100 µm.

Previous work has shown that cell cycle exit of hair cells in the canal cristae starts around E11.5 [44]. Accordingly, *Prox1* expression is detected prior and during cell cycle exit of hair cells and supporting cells of the canal cristae (Fig. 1). In contrast, in the cochlea *Prox1* expression started to be detected in the cells of the apex at around E13.5; although, it was faintly expressed in cells near the base at this stage (Fig. 4A) and clearly is upregulated only after hair cells have exited the cell cycle [40].

**Figure 5:**
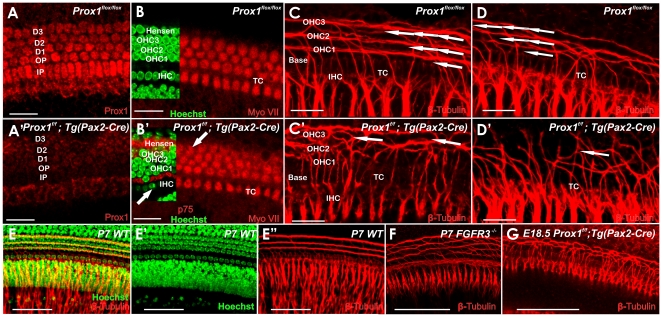
Fiber growth to outer hair cells is defective in E18.5 Prox1^ flox/flox^; Tg(Pax2-Cre) mutant embryos. A. Prox1 antibody staining reveals the normal expression of pattern of Prox1 in supporting cells (three rows of Deiter's cells, D1–3; two rows of pillar cells, IP, OP). There is also faint immunostaining in cells medial to the inner pillar cell (IP), probably in inner phalangeal cells. A′. Successful conditional inactivation of Prox1 is indicated by the barely detectable expression of Prox1 in *Prox1^ flox/flox^*; Tg(*Pax2-Cre)* mutant littermates at this stage. B, B′. Wildtype mice have four rows of hair cells (three rows of outer hair cells, OHC1–3, one row of inner hair cells, IHC). As seen by Myo VII staining, a partial fourth row and some misaligned outer hair cells (arrows) were occasionally detected in *Prox1^ flox/flox^*; Tg(*Pax2-Cre)* mutant embryos that is obvious in Hoechst stain with p75 labeling of pillar and Hensen cells (inserts). Otherwise, no other obvious changes in the distribution and maturation of Myo VII-expressing hair cells were detected. C, D. Normally and as seen by β-tubulin immunostaining, fibers grow out through the tunnel of Corti (TC) and turn to form three parallel outer spiral bundles (arrows) that run along Deiter's cells to reach the three rows of outer hair cells (OHC) in the base. C′, D′. Guiding defects in the extension of these fibers to outer hair cells are obvious in conditional *Prox1^ flox/flox^*; Tg(*Pax2-Cre)* mutant littermates in the middle turn. In this case, fibers follow a predominantly radial path with random turns toward the apex and the base. Further comparison with wildtype (E, E′, E″) and FGFR3 null mutant mice (F) shows the level of disorganization more clearly (G). FGFR3 mutants have disorganized supporting cells much like the *Prox1*-null mice but clearly do not show an equally severe disorganization of afferent growth (compare F with G. Bar, 20 µm.

Multiple rows of hair cells and supporting cells form initially as a short aggregate, but undergo convergent-extension movement to eventually form three rows of outer and one row of inner hair cells [49]. At around this stage of convergent extension, hair cells have already exited the cell cycle which progresses from the apex to the base of the cochlea between E11.5–E14.5 [40], [44], [45]. X-gal staining of *Prox1^+/LacZ^* embryos and *Prox1* in situ hybridization at different developmental stages revealed that in the cochlea, *Prox1* expression progressed initially from the apex to the base (Fig. 1B,C; Fig. 4A); a result suggesting that its expression is in cells that have already exited the cell cycle [45]. As shown in Fig. 4B–D, by E17.5, *Prox1* expression is prominent throughout the cochlea and near the base is almost exclusively detected in the five supporting cells of the lesser epithelial ridge (the three rows of Deiter's cells and the two rows of pillar cells); only limited expression was seen in some outer hair cells and inner phalangeal cells (arrows in Fig. 4D, D′). This limited expression in inner phalangeal cells seen in the X-gal stained and photoactivated organ of Corti (Fig. 4C′, D′), is also observed when using Prox1 antibodies (Fig. 5A). At this stage, *Prox1* expression in the apex is fainter and not organized into the five rows of supporting cells (Fig. 4E, E′; [29]. The prominent expression in supporting cells remained during postnatal stages, at least until P16 as shown by X-gal staining of *Prox1^+/LacZ^* (Fig. 4F,G; [29]. In later stages, a faint Prox1 signal was also detected in sensory neurons (Fig. 4F). This signal was more prominent using *in situ* hybridization (Fig. 4H,I). We verified the expression of Prox1 as revealed by X-gal staining using in situ hybridization (inserts in Fig. 4A, C; Fig. 4H,I0 and immunocytochemistry. For unknown reasons, X-gal staining of *Prox1^+/LacZ^* was easily lost after fixation in sensory neurons and could be demonstrated only in unfixed ears (Fig. 1D and insert). We also verified the supporting cell and neuronal expression that was so obvious with in situ hybridization starting at E15.5 (Fig. 4A, insert; Fig. 4C, insert) with immunocytochemistry (Fig. 4J). Combined, all three techniques show a profound upregulation of Prox1 in supporting cells and sensory neurons (with the caveat of suppression of X-gal staining of *Prox1^+/LacZ^* in sensory neurons following fixation).

**Figure 6:**
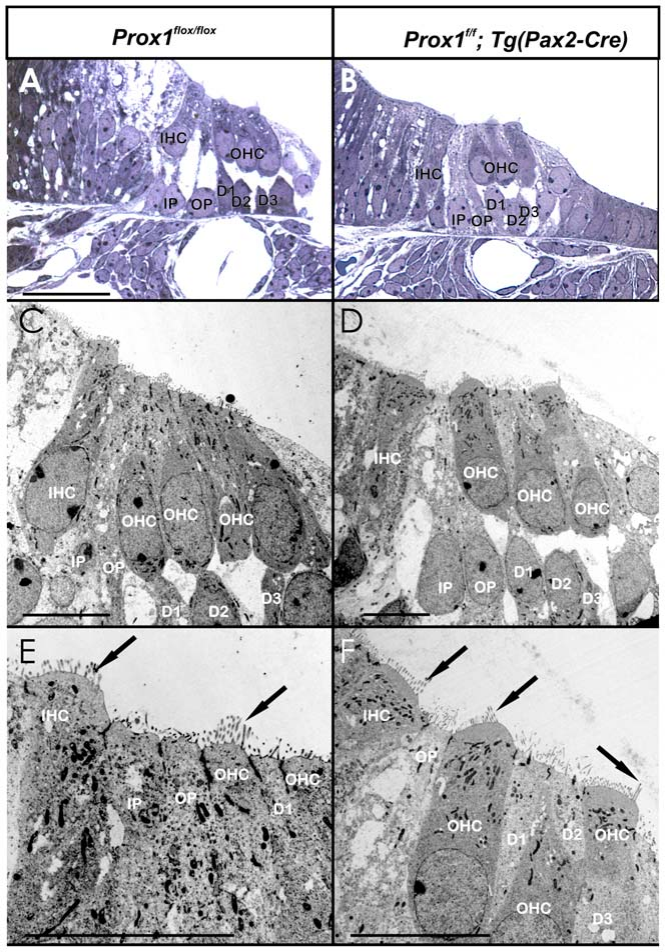
Organization of hair cells and supporting cells is mildly disrupted in Prox1^ flox/flox^;Pax2-Cre conditional mutant embryos. This image shows near radial thick (A,B) and ultrathin (C–F) sections through the middle turn of a *Prox1^flox/flox^* control and a *Prox1^flox/flox^*; *Pax2-Cre* conditional mutant animal. Note that the overall organization into 4 rows of hair cells (one inner and three outer) and five rows of supporting cells surrounding outer hair cells (two rows of pillar and three rows of Deiter's cells) is preserved in the conditional mutant (B,D,F). However, closer examination reveals that the regular organization of hair cells and supporting cells with two heads of pillar cells between inner and first row of outer hair cells (A,C,E) is only partially conserved in conditional mutants. In fact occasionally only a single pillar cell is found between inner and outer hair cells that appears to be the outer pillar cell (D,F). Hair cells develop normal with respect to apical kinocilia and stereocilia polarity and development (arrows in E,F). Abbreviations: D1–D3, first to third row of Deiter's cells; IHC, inner hair cell; IP, inner pillar cell; OHC, outer hair cell; OP, outer pillar cell. Bar indicates 100 µm in A,B and 10 µm in D–F).

In order to determine whether *Prox1* expression in supporting cells (Fig. 4C,D, D′, J,K,L′) is an indication that its functional activity is required to control any developmental aspect of this cell type, we analyzed the cochlea of E18.5 *Prox1^f/f^;Tg(Pax2-Cre)* mutant embryos. Using this approach, *Prox1* expression was extensively removed from the developing cochlea (Fig. 5A′). Although as discussed above (Fig. 2D, F), no obvious alterations in hair cell differentiation were observed, hair cells patterning was found to be occasionally disrupted. At this stage and as shown by Myo VII staining (Fig. 5B), wild-type hair cells exhibit the typical one row of inner hair cells and three parallel rows of outer hair cells. In the mutant littermates, inner and particularly outer hair cells appeared disorganized, misaligned, and containing extra rows near the apex (Fig. 5B′arrows). These results indicated that lack of Prox1 function did not affect hair cell differentiation (hair cell differentiation markers Myo VII and BDNF were normally expressed in the mutant hair cells; Figs. 5B, B′, 6); however, hair cell patterning was slightly defective. Light and electron microscopic radial sections confirmed the near normal development of hair cells and supporting cells but also some degree of disorganization of both cell types (Fig. 6).

**Figure 7:**
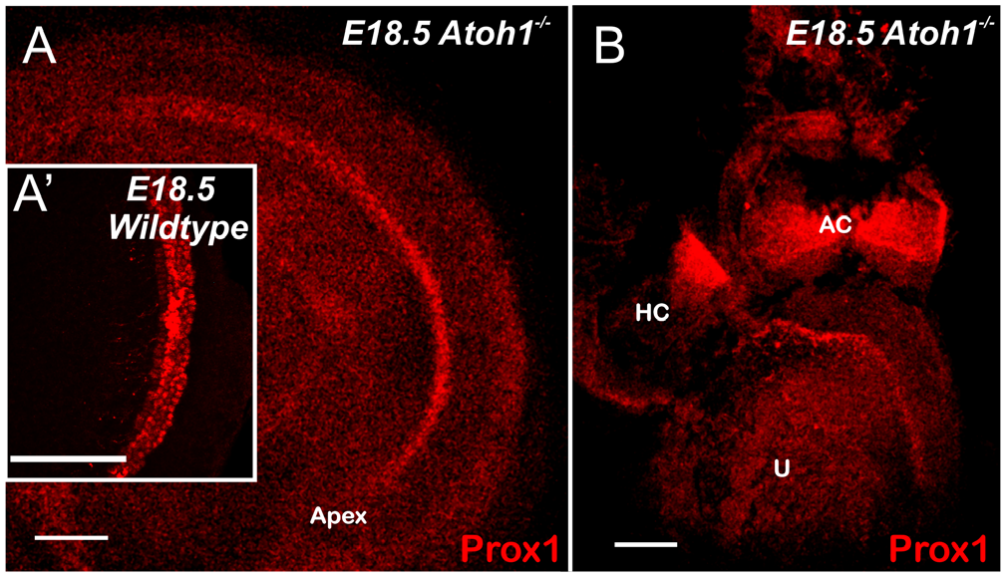
Hair cell differentiation is not required for Prox1 expression. A, B. Prox1 expression is maintained in undifferentiated supporting cells of E18.5 *Atoh1*- null embryos. This result argues that *Prox1* expression is independent of hair cell mediated differentiation of sensory epithelia. A′ shows the Prox1 immunostaining in the apex. Abbreviations: AC, anterior crista; HC, horizontal crista; U, utricle. Bar, 100 µm.

These results indicate that Prox1 activity is not required for hair cell differentiation were further corroborated by the fact that Prox1 expression was not affected in E18.5 *Atoh1*-null embryos (Fig. 7) with defective hair cell differentiation [37], [50]. These results also demonstrate that *Prox1* expression is not dependent on *Atoh1* or on hair cell differentiation consistent with recent reports, indicating autonomy of Prox1 expression from hair cell differentiation [4], [51].

Together, these results suggested that in the organ of Corti, lack of *Prox1* function promotes some limited phenotypic alterations in the overall patterning resulting in a slightly disorganized distribution of supporting and hair cells, including short extra rows of outer hair cells and misalignment of inner hair cells. Interestingly, direct comparison of *Prox1^flox/flox^;Tg(Pax2-Cre)* and wildtype littermates showed that the Prox1 *in situ* signal was somewhat weaker in the organ of Corti but unaltered in the sensory neurons (Fig. 4H,I). This could indicate that the disorganization of supporting cells may affect overall level of Prox1 expression.

**Figure 8:**
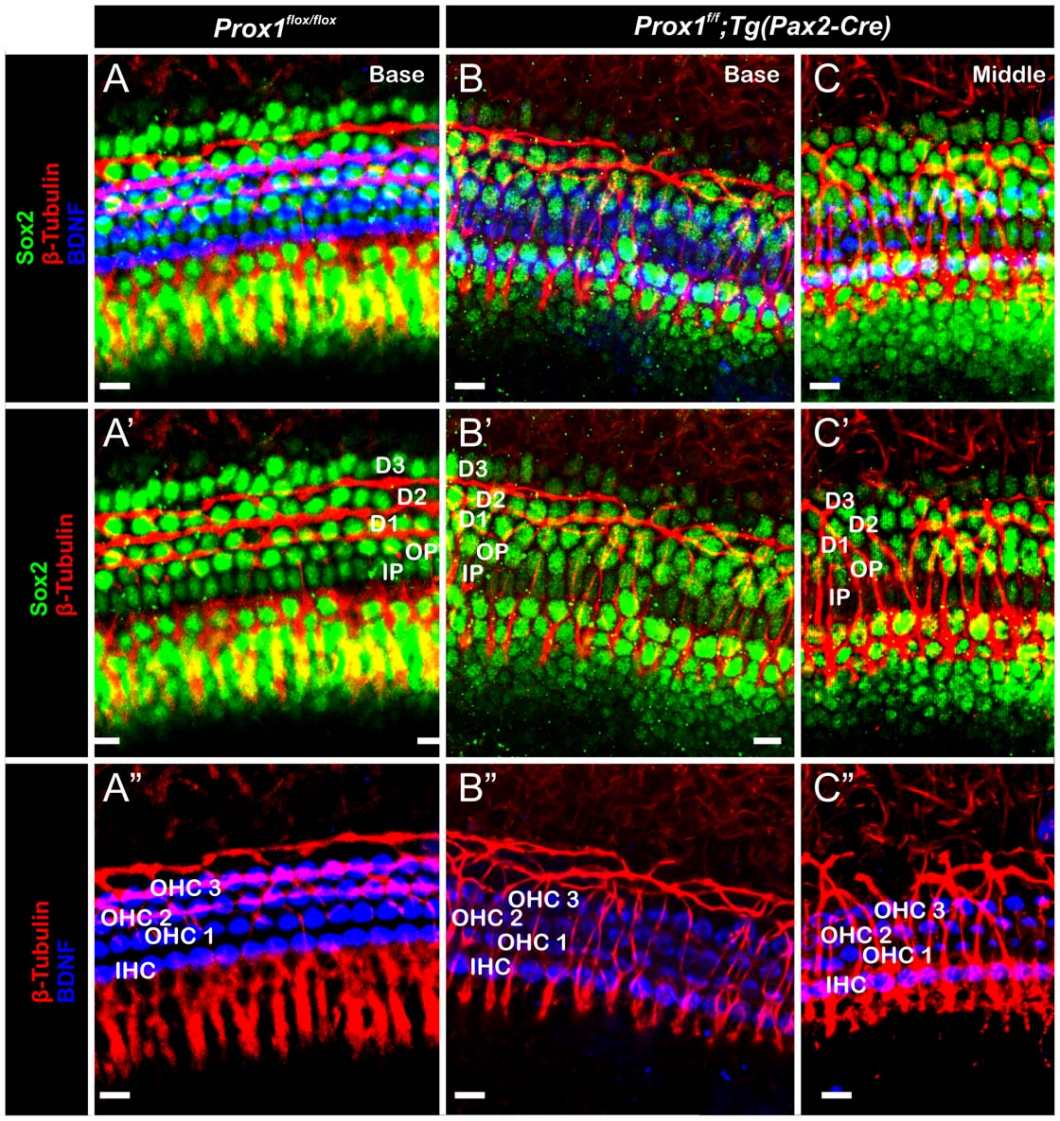
Triple immunolabeling reveals cellular and fiber disorganization in the organ of Corti of Prox1^ flox/flox^; Tg(Pax2-Cre) conditional mutant embryos. Whole mount antibody staining of the organ of Corti highlighting the hair cells (anti-BDNF, blue), supporting cells (anti-Sox2, green) and nerve fibers (anti-β-tubulin, red). (A–C) The top row shows all three immunostaining together, the middle shows nerve fibers and supporting cells, and the bottom one nerve fibers and the hair cells. In contrast to the wild-type condition (A, A′ A″), in *Pax2-Cre;Prox1^ flox/flox^* conditional mutant embryos fibers extend beyond the first row of Deiter's cells (B′,C′) where they turn randomly toward the base or apex. In addition, hair cells are not in close proximity to the nerve fibers (A″, B″, C″). D1–D3− Deiter's cells, IP-inner Pillar cell, OP-outer Pillar cell, IHC-Inner hair cell, OHC1–3-outer hair cells. Bar, 100 µm.

**Figure 9:**
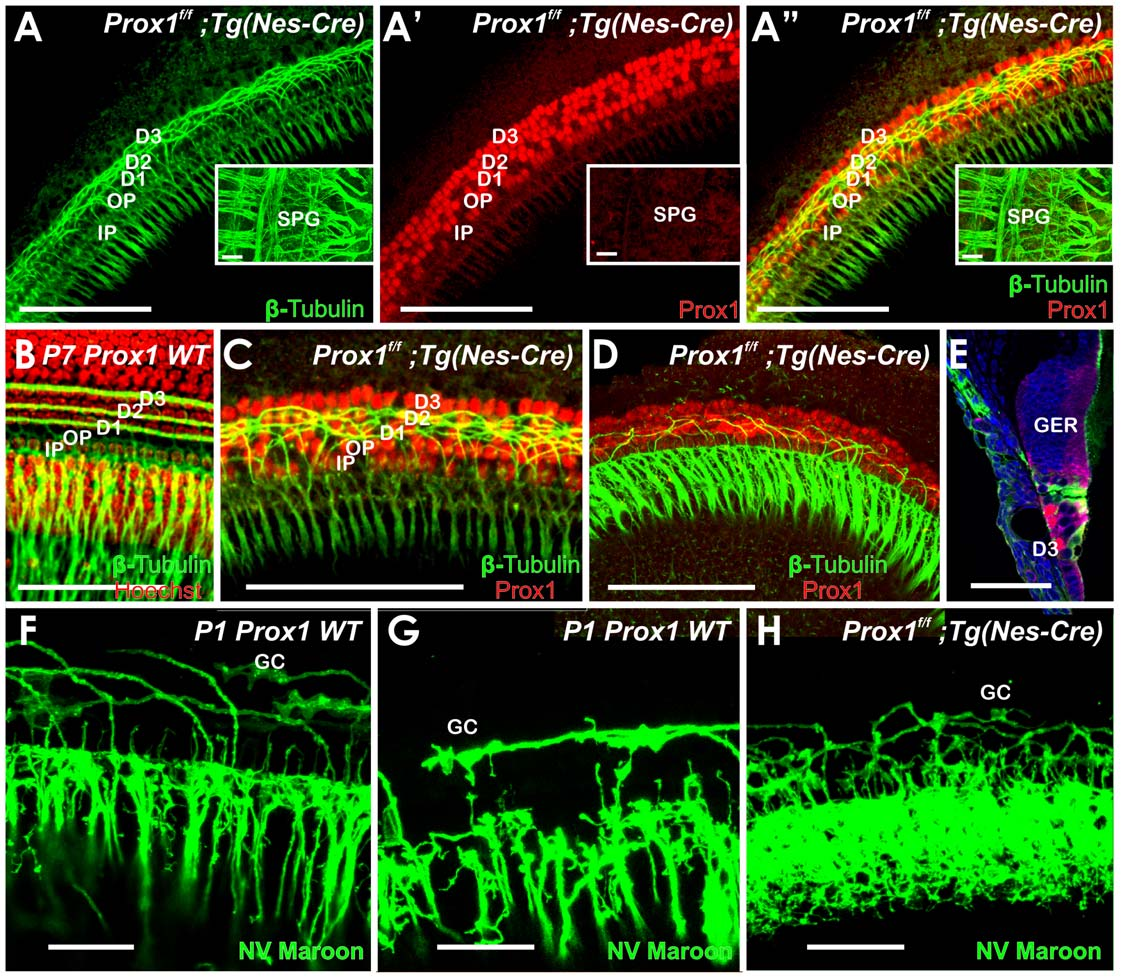
Immunolabeling and dye tracing reveals Type II fiber disorganization in the organ of Corti of Prox1^ flox/flox^; Tg(Nes-Cre) conditional mutant embryos. (A, A′,A″) This 1 day old basal turn shows that the Prox1 protein is present in the supporting cells (A′, A″) and that neuron processes extend beyond the first row of Deiter's cells to form a bundle of intertwined fibers near the second and third row of Prox1 positive Deiter's cells. Inserts in A, A′ and A″ show tubulin immunostaining in spiral ganglion cells (SPG) but show no immunoreaction for Prox1. The disorganization of nerve fibers becomes particularly obvious in a side by side comparison with the regular pattern of cells (shown with Hoechst stain) Type II process in wildtype (B,C). In the apex, Type II fibers extend in a random way towards base and apex between Prox1 positive supporting cells (D). Epoxy section of Prox1 (red) and tubulin immunostained (green) and Hoechst counterstained (blue) organ of Corti shows the normal organization of the greater epithelial ridge (GER) with Prox1 being restricted to 5 rows of supporting cells. Point applications of lipophilic dyes allows imaging the growth cones and their regular turns toward the base in wildtype (F,G) but shows a disorganized outgrowth and growth cones (GC) in *Prox1^ flox/flox^*; *Nes-Cre* conditional mutant mice. D1–3, Deiter's cells row 1–3; IP, inner pillar; OP, outer pillar; SPG, spiral ganglion. Bar, 100 µm (A–D), 50 µm (E–H; inserts in A,A′, A″).

Next, we analyzed whether these phenotypic alterations identified in supporting and hair cells affected nerve fiber growth. Previous work [52], [53], [54], [55], [56] showed that the stereotyped growth of Type II fibers toward outer hair cells is more advanced in the base and upper middle turn (Fig. 5C, D). Type II spiral ganglion cells extend first radially through the tunnel of Corti and then turn sharply toward the base to form three parallel rows in front of the three rows of Deiter's cells that are spaced between the three rows of outer hair cells (arrows Fig. 5C, D). We found that in *Prox1^flox/flox^; Tg(Pax2-Cre)* mutant littermates, the fibers also extended radially (arrows Fig. 5C′, D′). We found that all mutant nerve fibers extended beyond the first row of Deiter's cells (D1; Fig. 8B′, B″) and randomly turned at the 2^nd^ and 3^rd^ row of Deiter's cells (D2, D3; Fig. 9C,C′, C″). No obvious reduction in the density of the radial fibers was observed (Fig. 5C′, D′). These results were corroborated further by triple immunolabeling where supporting cells were identified by the use of Sox2 antibodies (Fig. 8). The normal organization of the Sox2-expressing supporting cells (green), BDNF-expressing hair cells (blue), and β-tubulin-expressing fibers (red) is shown in Fig. 8A–A″. In contrast, misaligned supporting and hair cells are seen in E18.5 *Prox1^flox/flox^;Tg(Pax2-Cre)* mutant littermates (Fig. 8B–B″, C–C″). These results and those obtained using electron microscopy (Fig. 7) suggested that in the absence of *Prox1*, the signaling mechanisms controlling where and to which direction fibers should turn is disrupted. Close comparison between wildtype (Fig. 5E), FGFR3 null mice (Fig. 5F) and E18.5 *Prox1^flox/flox^;Tg(Pax2-Cre)* show that type II afferents are disorganized in FGFR3 null mice (Fig. 5E.F), but that this disorganization is different from that seen in *Prox1^flox/flox^;Tg(Pax2-Cre)*.

Next we investigated fiber growth in *Prox1^flox/flox^;Tg(Nes-Cre)* mice to evaluate possible spiral sensory neuron cell autonomous defects. Nestin (*Nes*), a neuronal stem cell marker, is expressed in developing sensory neurons of the ear [57]. We used a *Tg(Nes-Cre)* line [36] to conditionally delete *Prox1*. As seen in Fig. 9A, Prox1 expression was deleted in the spiral neurons but remained in the sensory epithelium (Fig. 9A, A′, A″). We traced the nerve fibers with lipophilic dyes or tubulin immunocytochemistry. Similar to what was observed in *Prox1^flox/flox^;Tg(Pax2-Cre)* pups, P1 *Prox1^flox/flox^;Tg(Nes-Cre)* mice showed severe disruption in the organization of Type II fibers (Fig. 9A,C,D,H). In the apex, where Type II fibers are growing out we could clearly identify that each fiber made an almost random turn to either the base or the apex (Fig. 9D,H) compared to the stereotyped decision of growth cones in wildtype (Fig. 9F,G), which always turn toward the base. Near the base, were Type II fibers have a longer trajectory at this stage, we find an intertwined mesh of fibers near the second and third row of Deiter's cells (Fig. 9C) instead of the very regular organization near all three rows of Deiter's cells (Fig. 9B). Given that our Prox1 antibody shows a clear, only somewhat interrupted signal in supporting cells (Fig. 9A′, A″, C,D), it seems that this disruption of fiber projection is predominantly due to the lack of Prox1 expression in sensory neurons in the *Prox1^flox/flox^;Tg(Nes-Cre)* conditional null mice, a signal which, according to our *in situ* hybridization data, is becoming increasingly prominent after E14.5 (Fig. 4H,I). Since the first Type II fibers are growing toward outer hair cells at around E16.5 [53], [55], it appears that Prox1 upregulation coincides with the ability of Type II fibers to navigate their normal stereotyped trajectory. In the absence of Prox1 either in sensory neurons or in sensory neurons and supporting cells combined this ability is partially disrupted. However, Type II fibers may be able to reach the outer hair cells but extend beyond the first row of Deiter's cells thus might miss the first row of outer hair cells.

**Figure 10:**
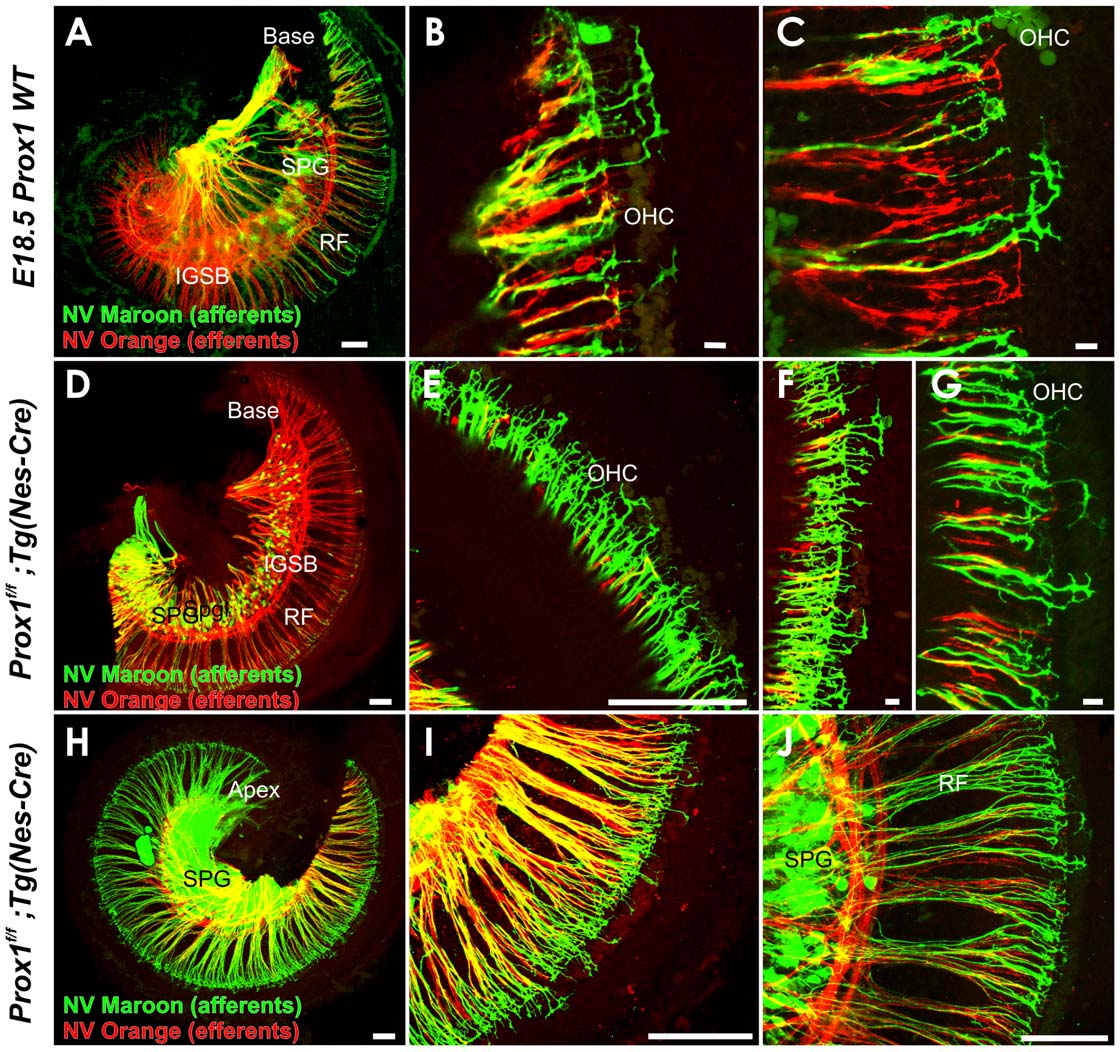
Dye tracing reveals Type II fiber outgrowth problems in the organ of Corti of Prox1^ flox/flox^; Tg(Nes-Cre) conditional mutant embryos. NV Maroon (green) was inserted into the cochlear nucleus and NV Orange (red) was inserted into the olivocochlear bundle to label a small population of afferents (green) and all efferents (red). Efferents show a similarly organized intraganglionic spiral bundles in wildtype (A–C) and *Prox1^ flox/flox^*; *Nes-Cre* conditional mutant mice (D–J) and grow toether with afferents in radial fiber bundles (RF) to the organ of Corti. Note that at this stage only occasional efferents extent to outer hair cells. In contrast, type II afferents grow to the second or third row of outer hair cells (OHC) where they invariably turn toward the base (B,C). At this stage, none of the multiple type II afferents of *Prox1^ flox/flox^*; *Nes-Cre* conditional mutant mice show this coordinated growth pattern. Instead, fibers grow randomly toward the base or apex but mostly seem to stall with multiple branches extending toward the base and the apex (F,G). IGSB, intraganglionic spiral bundle; OHC, outer hair cells; RF, radial fibers; SPG, spiral ganglion. Bar, 100 µm (A–D), 50 µm (E–H; inserts in A,A′, A″).

To better understand the inability of type II fibers to turn correctly, we investigated the outgrowth of fibers to the outer hair cells in E18.5 *Prox1^flox/flox^;Tg(Nes-Cre)* conditional null mice (Fig. 10). We used different colored lipophilic dyes to trace small subsets of spiral sensory neurons from the cochlear nuclei [52]. To avoid confusion with the second fiber type that reaches the outer hair cells, the olivocochlear efferent system [58], [59], we labeled these fibers with a differently colored lipophilic dye [60]. At this stage, only type II afferents grow to outer hair cells. In control mice all fibers navigated their way between supporting cells and turned invariably toward the base (Fig. 10. A–C). In contrast, in *Prox1^flox/flox^;Tg(Nes-Cre)* conditional null mice we found that the initial fiber growth was undirected, frequently stalled with branches in both directions or turned randomly toward the base or the apex (Fig. 10D–J). Absence of Prox1 protein disables recognition of directional signals during type II fiber growth.

## Discussion

In this paper we identified *Prox1* as a novel regulator of inner ear development and extent previous expression data [28], [29]. We determined that this gene's activity is required for the proper growth of the canal cristae and correct fiber patterning of Type II afferents in the cochlea. Consistent with the transient low level of expression, no phenotypic alterations were identified in the gravistatic receptors organs (utricle and saccule).

### Prox1 Regulates Canal Cristae Growth

During inner ear development, one of the earliest and more prolonged expression patterns of *Prox1* was detected in the canal cristae. In this organ, the onset of *Prox1* expression overlaps with that of other gene products such as *Gata3* [61], *Fgf10* [62], *Foxg1* [63], *Sox2* [64], *Lmx1a* [65] and *Bmp4* [66], whose activities are essential for the formation and differentiation of the sensory epithelia. In general, gene inactivation of any of these factors resulted in the partial or total loss of the sensory canal cristae [67], [68] or overgrowth [65]. In *Prox1*-null embryos the canal cristae did not exhibit any morphological alteration; however, their size was reduced. Accordingly, it could be speculated that *Prox1* activity is necessary to maintain and expand the pool of neurosensory progenitor cells. *Atoh1* is essential for hair cell differentiation [50] and *Atoh1*-null mice fail to differentiate hair cells and supporting cells [37]. Therefore, our finding that *Prox1* expression remained normal in *Atoh1*-null ears, and that *Prox1*-null hair cells expressed typical hair cell markers eliminates the possibility that *Prox1* was required for hair cell differentiation at the level of neurosensory progenitors. This does not rule out that misexpression of Prox1 in hair cells can result in their degeneration, as was recently shown for cochlea but not for vestibular hair cells [29].

### Prox1 Regulates Fiber Guidance of Type II Spiral Neurons in a Cell Autonomous Way

Similar to what has been reported for the cell cycle kinase inhibitor *p27* [45], [69], the neurotrophin *Bdnf* [70], [71] and the growth factor *Fgf10* [62], *Prox1* expression in the cochlea starts to be detected almost a day after hair cell precursors exited the cell cycle [40], [45]. While Prox1 is not expressed in hair cell progenitor cells, it is expressed transiently in differentiating hair cells [29]. However, its continued expression in organ of Corti cells of *Atoh1*-null mice [4], [51], who have only hair cell precursors that fail to differentiate [37], indicates that at least the expression in supporting cells is not regulated by Atoh1 or other genes specifically expressed in differentiated hair cells (Fig. 7). Given that Prox1 expression persists at least until P26 in supporting cells [29], it is possible that this gene remains expressed after at least neonatal hair cell loss and its promoter could be used to drive molecular expression toward reconstitution of the a functional organ of Corti.

As previously reported [28], later during embryogenesis *Prox1* expression is detected in the five supporting cells of the lesser epithelial ridge (Fig. 4). In these cells, lack of *Prox1* function lead to subtle phenotypic alterations; e.g., defective alignment of hair cells and supporting cells (Fig. 5) However, major pathfinding defects were identified in Type II spiral ganglion fibers. In this case, the turning of these fibers toward the base [52], [55] was severely disrupted (Fig. 5,8,9,10). We found that in conditional null mutants fibers abnormally extended toward the second and third rows where they turned randomly instead of turning toward the base in front of each of the three rows of Deiter's cells. Radial fiber growth beyond the inner pillar cells was not affected. It is worth mentioning that pathfinding defects have been identified in the CNS of *Prospero* mutant flies [72].

While *Prox1* is the first gene that plays a cell autonomous role in Type II pathfinding, at the moment it is not known how Prox1 affects fiber pathfinding of these neurons. It is known that *Fgf8* and *Fgf10* mediated activation of *Fgfr1*, *2b* and *3* signaling participates in the differentiation of supporting cells of the lesser epithelial ridge [3], [62], [73], [74], [75], [76], and *Fgfr3* -null mice also exhibit short extra rows of outer hair cells [3], [77] with some minor fiber disorganization that is clearly distinct from the Prox1 effects (Fig. 5F,G), but where exactly *Prox1* fits into these interactions remains to be determined.
